# Insight into the feeding behavior of predatory mites on *Beauveria bassiana*, an arthropod pathogen

**DOI:** 10.1038/srep24062

**Published:** 2016-04-04

**Authors:** Shengyong Wu, Ye Zhang, Xuenong Xu, Zhongren Lei

**Affiliations:** 1 State Key Laboratory for Biology of Plant Diseases and Insect Pests, Institute of Plant Protection, Chinese Academy of Agricultural Sciences, Beijing 100193, P.R. China; 2 Shanxi Key Laboratory of Integrated Pest Management in Agriculture, Institute of Plant Protection, Shanxi Academy of Agricultural Sciences, Taiyuan, Shanxi, 030031, P.R.China

## Abstract

Interactions between fungal entomopathogens and pest predators are particularly relevant in control of agricultural insect pests. In a laboratory study, we confirmed that the predatory mite, *Neoseiulus barkeri*, exhibited feeding behavior on the entomopathogenic fungus *Beauveria bassiana* conidia through DNA extracts. Using transmission electron microscopy, we determined that the majority of conidia found in the mite gut tended to dissolve within 24 h post ingestion, suggesting that the conidia had probably lost their viability. To our knowledge this is the first report of feeding behavior of phytoseiid mites on entomopathogenic fungus. The findings expand our knowledge of fungus–predator interactions.

The use of multiple natural enemies has been recommended to control insect pests in integrated pest management programs^1^ although it remains controversial whether the pest control is more beneficial from the release of single or multiple natural enemies into agroecosystems. Multiple enemy species may interact synergistically in pest populations, thereby increasing their control efficacy^2^,^3^. On the other hand, they may interfere with each other due to negative interspecific interactions, resulting in reduced efficiency in pest control^4^. A better understanding of the interactions between different natural enemies would be of primary importance in integrated pest management programmes.

The entomopathogenic fungus *Beauveria bassiana* is known to be effective in controlling many arthropod pests^5^. Microbial control of pests is an important approach to reducing dependence on chemical pesticides for increased agricultural sustainability. Conventional spray of a fungal formulation is often applied to control the pests that can be readily contaminated with deposited conidia, such as foliage dwellers. However, it is difficult for the fungal spray to contact the pests inside flowers or on the undersides of leaves. In such cases, the introduction of other natural enemies, such as predators, becomes necessary to control the hiding pests. Predatory mites are often used as an alternative to conventional pest management on a variety of plants^6^. Since *B. bassiana* has shown to have no pathogenicity to the predatory mite *Neoseiulus barkeri* (Acarina: Phytoseiidae)^7^, this predator is considered to be biologically compatible with the fungal insecticide.

Most previous studies on the interactions between fungal pathogens and predatory mites have focused on the fungal infectivity to predators^8^,^9^, or on the sublethal effects of ingesting pathogen-treated prey on predators^10^,^11^. However, predatory mites may also have an opportunity to ingest the fungal conidia deposited on leaves or even on prey bodies following spray application. Fungal grazing by mite species^12^,^13^, is fairly common in nature. Fungi have also been reported as an alternative food source of several predatory mites, including *N*. *barkeri*^14^, *Typhlodromus pyri*^15^ and *Amblyseius swirskii*^16^, although those fungi are primarily plant or soil inhabiting fungi. However, predatory mites feeding on entomopathogenic fungi have not been previously studied.

This study seeks to investigate feeding behavior of *N. barkeri* on *B. bassiana*. In addition, ingested *B. bassiana* conidia inside the mite gut were morphologically compared with normal to reveal the viability and pathogenicity of conidia as well as transmission efficiency after ingestion.

## Results

**Figure 1:**
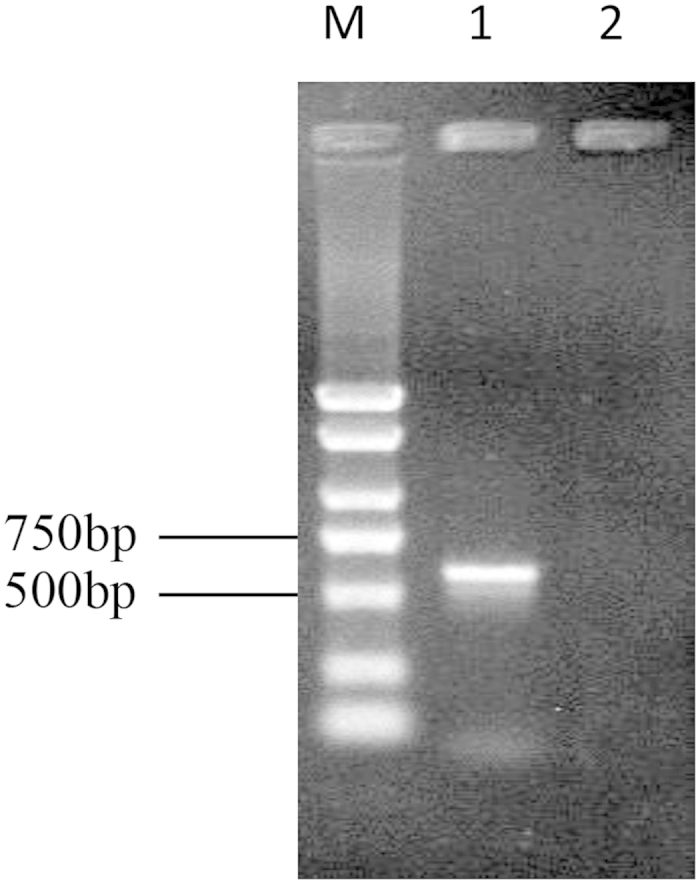
PCR detection of *B. bassiana* ITS in the intestine of *N. barkeri*. Sample Loading: (M) DNA Marker; (1) *B. bassiana* – fed mites; (2) untreated mites.

After *B. bassiana* inoculation, conidia were ingested by *N. barkeri* through long narrow esophagus for entry into broad midgut under a microscope (see Supplementary Video S1). Fungal DNA was subsequently extracted from the guts of the treated mites, contrasting to no fungal DNA signal detected in the extract from untreated mites (Fig. 1). A BLAST search indicated that the ITS sequence in the fungal DNA extract showed a high similarity with those of other strains of *B. bassiana* (>95%). Phylogenetic analysis showed that it was closely related to the Bb3113 strain.

**Figure 2:**
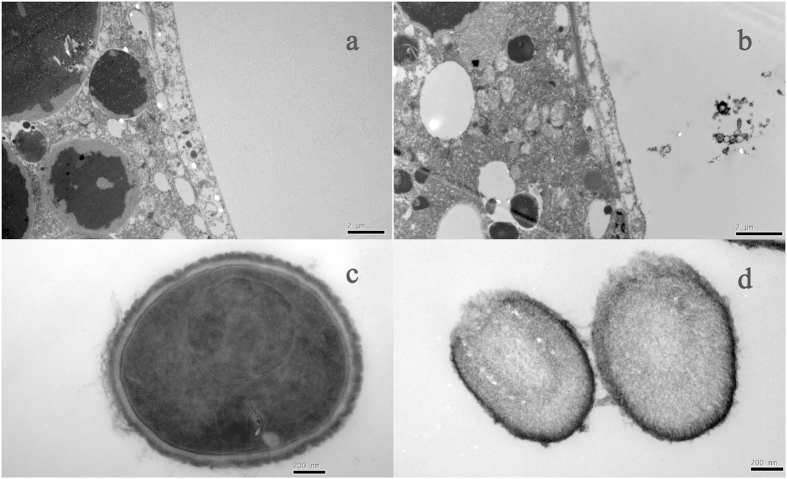
TEM image of intestine of *N. barkeri* and *B. bassiana* conidia. (**a**) The intestine of starved mite; (**b**) The intestine of *B. bassiana* – fed mite; (**c**) Normal conidia with intact cell wall and chromatin; (**d**) Conidia with dissolved cell wall in the intestine of mite 24 h after treatment with *B. bassiana*.

As demonstrated by transmission electronic microscopy (TEM), residual conidia were observed in the intestines of treated mites, but not in the untreated mites (Fig. 2). Furthermore, cell walls of the majority of conidia in the gut of treated mites tended to dissolve, compared to the intact cell walls of uningested conidia.

## Discussion

Compatibility between entomopathogenic fungi and predatory mites is of critical importance for controlling their shared pests. For example, predators may also function as dissemination agents in exposing susceptible hosts to conidia leading to infection in the pest species^17^. Conversely, if the fungal transmission of conidia is interfered with the predators or if a negative effect exists between predators and fungi, the overall efficacy of fungi to control target pest species will be affected. In this study, the impaired cell walls of ingested conidia in the intestine of mites suggest that the conidia could have lost their viability and infectivity. Germination of *B. bassiana* conidia depends on the water activity of the nutrient liquid^18^. Under normal nutritional and environmental conditions, most conidia may germinate within 18 h^19^. The dissolved conidia found in the mite intestines 24 h after ingestion suggests that these conidia are unable to germinate after excretion. It is unclear why the degradation occurs due to the lack of information on the digestive system and the nature of the liquid environment in the gut of phytoseiid mites.

The pathogenicity of a fungus to a host insect is mainly caused by the conidia penetrating the insect’s cuticle^20^. While our previous study demonstrated that geminated conidia of *B. bassiana* were not able to penetrate the cuticle of *N. barkeri*^7^, the present study provides complementary evidence that *B. bassiana* has no pathogenicity to *N. barkeri* through ingestion. The *B. bassiana*–fed mites became smaller in body size after 24 h and more transparent within 48 h compared to normal mites fed with mite prey. These observations implicate that the conidia ingested by the mites were of poor nutritional value.

Predator-fungus interactions are complex. They can interact directly through conidia dissemination with positive consequences in suppressing a pest population. Conversely, they may interact indirectly mediated by their common prey or fungal conidia causing antagonistic effects. The present study provides new examples of negative interactions between *B. bassiana* and predatory mites. That is, the feeding behavior of *N. barkeri*, on *B. bassiana* conidia would reduce the amount of sprayed conidia should the two control agents be presented simultaneously in an agroecosystem.

## Methods

### Ethics Statement

No specific permissions were required for these locations/activities.

None of the species used in this study are endangered or protected.

### Fungal preparations

The isolate of *B. bassiana* SZ-26 was derived from *Ostrinia nubilalis* (Lepidoptera: Pyralidae) larvae collected in Suizhong, Liaoning, China (2010). Fresh cultures were maintained on Sabouraud dextrose agar (SDA) for conidiation at 26 ± 1 °C under continuous darkness. Conidial concentration (1 × 10^8^ conidia mL^−1^) was prepared with sterile 0.05% Tween-80. Viability was confirmed on SDA^21^, and the germination percentage of the conidia was determined to be >90%. The strain SZ-26 was chosen because of its high virulence to the western flower thrips *Frankliniella occidentalis*, but no infectivity to *N. barkeri*^7^.

### Predatory mite colony

A colony of *N. barkeri* was maintained in the Laboratory of Insect Natural Enemies, Institute of Plant Protection, Chinese Academy of Agricultural Sciences. *N. barkeri* stock colonies were cultured on excised kidney bean (*Phaseolus vulgaris*) leaves in a covered plastic container (15 cm × 15 cm × 10 cm)^22^, and provided with *Tyrophagus putrescentiae* as prey. The newly emerged adult females were used in the feeding experiments.

### Detection of feeding behavior of *N. barkeri* on fungal conidia

Experiments were conducted using two uniform pieces of plexiglass (6 cm × 5 cm × 4 mm). A water-saturated filter paper was placed on one piece of plexiglass, and a freshly excised kidney bean leaf disc was placed topside down on the surface of the filter paper. A 2.5 cm diameter hole was punched in another piece of plexiglass and placed on top of the leaf, forming a feeding and observation chamber between the two pieces of plexiglass. Prior to placing the second piece of plexiglass, a *B. bassiana* suspension was sprayed into each chamber using a 2 ml hand pressure sprayer. Once the conidia were deposited on the leaf, 10 *N. barkeri* mites were transferred into each feeding chamber and the second piece of plexiglass was placed on top. The layers were tightly clipped together on both ends to avoid mites escaping. The feeding behavior of mites on the *B. bassiana* suspension was observed under a microscope. To analyze the conidia in the gut of mites, the mites were allowed to feed on the *B. bassiana* for 30 minutes. They were then removed and the mites were eluted with sterile 0.05% Tween-80 to remove any residual conidia adhering to the surface of the mites. Ten mites were then manually homogenized in liquid nitrogen and the DNA extracted according to the manufacture’s instruction using the Plant Genomic DNA Kit (Tiangen Biotech (Beijing Co., Ltd). The internal transcribed spacer (ITS) was amplified and sequenced with universal primers ITS4 (TCCTCCGCTTATTGATATGC) and ITS5 (GGAAGTAAAAGTCGTAACAAGC)^23^. To further verify that the fungal DNA was extracted from the interior of mites rather than from their exterior surface, the control consisted of mites sprayed with a *B. bassiana* suspension and then immediately eluted with sterile 0.05% Tween-80. Fungal DNA was extracted as described above. DNA sequences were assembled and edited using Sequencher 4.1 (Sangon Biotech, Shanghai, China). The output DNA sequences were BLAST compared using NCBI and phylogenetic analysis was performed by MEGA.

### Transmission electronic microscopy observations

To confirm the viability of conidia collected from the mite guts after 24 h exposure to *B. bassiana*, treated and untreated mites were fixed in 5% glutaraldehyde in a soda-cacodylate buffer at room temperature for 12 h. The mites were then rinsed five times with 70% ethyl alcohol, and dehydrated through an ascending series of ethyl alcohol (75, 80, 90, 95 and 100%, 6 min each) before embedding in Spurrs’ resin. Ultrathin sections from the intestine were cut and collected on carbon-stabilized Formvar-coated grids. The samples were then stained negatively with uranyl acetate solution and dried under silica gel. Thin sections were cut from the mite intestine using a microtome. The morphological structure of residual conidia from the gut of treated mites was observed via TEM and compared with non-ingested conidia. All the mite specimens were starved for 24 h prior to the test to minimize the influence of extraneous intestinal material.

## Additional Information

**How to cite this article**: Wu, S. *et al.* Insight into the feeding behavior of predatory mites on *Beauveria bassiana*, an arthropod pathogen. *Sci. Rep.* **6**, 24062; doi: 10.1038/srep24062 (2016).

## Supplementary Material

Supplementary Video S1 legend

Supplementary Video S1
