# Supplementary material for: Insight into the feeding behavior of predatory mites on Beauveria bassiana, an arthropod pathogen
Source: Sci Rep. 2016 Apr 4;6:24062. doi: 10.1038/srep24062 (PMC4819197; doi:10.1038/srep24062)
Supplement: Supplementary Video S1 legend [file srep24062-s1.doc]

**Supplementary Information**

**Insight into the** **feeding behavior of predatory mites on *Beauveria bassiana*, an arthropod pathogen**

Shengyong Wu^1*^, Ye Zhang^2^, Xuenong Xu^1^ and Zhongren Lei^1*^

^1^State Key Laboratory for Biology of Plant Diseases and Insect Pests, Institute of Plant Protection, Chinese Academy of Agricultural Sciences, Beijing 100193, P.R. China.

^2^Shanxi Key Laboratory of Integrated Pest Management in Agriculture, Institute of Plant Protection, Shanxi Academy of Agricultural Sciences, Taiyuan, Shanxi, 030031, P.R.China.

*Corresponding author E-mail: [wushengyong2014@163.com](mailto:wushengyong2014@163.com), zrlei@ippcaas.cn

**Supplementary Video S1** Feeding behavior of the predatory mite *Neoseiulus barkeri* on fungal conidia
